# Supplementary material for: Addressing a youth mental health crisis; utilising therapeutic landscapes and creative approaches in an english school
Source: J Outdoor Environ Educ. 2025 May 26;29(3):915–31. doi: 10.1007/s42322-025-00214-7 (PMC13739006; doi:10.1007/s42322-025-00214-7)
Supplement: Supplementary file 1 [file 42322_2025_214_MOESM1_ESM.docx]

**Title: Addressing a youth mental health crisis; Utilising therapeutic landscapes and creative approaches in an English school**

Clare Woolhouse, *Faculty of Education, Edge Hill University, Ormskirk, UK*

Catherine El Zerbi, *Faculty of Medical Sciences, Newcastle University, UK*

Charley McFarlane-Troy, *Faculty of Medical Sciences, Newcastle University, UK*

**Corresponding author:**

Dr Clare Woolhouse, Reader, Faculty of Education, Edge Hill University, St Helen’s Road, Ormskirk, Lancashire, L39 4QP, UK.

Tel: 01695 50 893, [Woolhouc@edgehill.ac.uk](about:blank)

Pure profile link: <https://research.edgehill.ac.uk/en/persons/clare-woolhouse>

ORCID : 0000 0002 2648 9997

**Biographies**

Dr Clare Woolhouse is currently a Reader in Inclusive at Edge Hill University. After completing her PGCE, Clare worked in two secondary schools and a further education college before moving into higher education. At Lancaster University she taught undergraduate students for five years while studying for a PhD. In 2007 she moved to the Faculty of Education at Edge Hill University. Her research adopts a feminist, post structuralist approach to exploring identity formation, multi-modal methodologies and pedagogies, with particular attention given to aspects of educational difference, inclusion, children’s rights and social justice utilising arts-based creative research methods. Further information can be found via her ORCID profile: <https://orcid.org/0000-0002-2648-9997>

Dr Catherine El Zerbi works as a mixed methods applied health researcher in the Population Health Sciences Institute at Newcastle University. She holds a PhD from King's College London. Further information can be found via her ORCID profile: <https://orcid.org/0000-0003-4041-2379>

Charley McFarlane-Troy has worked as a secondary school teacher for two years in China, then England after acquiring her MA in Anthropology and her PGCE in secondary English. Charley recently completed a research assistant role on the Rooted in Nature project and now works in a secondary school in Newcastle, UK as an English teacher.

**Acknowledgements**

We would like to thank the practitioners and young advisors who guided and supported the research as well as all the children and young people who were involved in the nature-based activities studied within the *Rooted in Nature* project.

We would also like to thank the Funder – the Arts & Humanities Research Council (Reference: AH/W008033/1). Dr Catherine El Zerbi is funded by the National Institute for Health and Care Research (NIHR) Applied Research Collaboration (ARC) North-East and North Cumbria (NENC) (NIHR200173). The views expressed are those of the authors and not necessarily those of the NIHR or the Department of Health and Social Care.

**Abstract**

This paper explores the Rooted in Nature research project, which developed a co-produced and arts-based methodology alongside children who were accessing nature-based activities designed to promote mental health and wellbeing. The aim of the project was to identify relevant health and social outcomes relating to involvement in these activities for the purposes of scaling up, particularly in the North East and North Cumbria in the UK.

Using a case study approach, this paper describes how the concept of therapeutic landscapes was utilised in the project and how the data set was gathered through the arts-based method of photo-elicitation with six pupils from a secondary school in North East England. The discussion of the findings focuses upon the views and experiences of the pupils involved in the activities and these are explored in terms of three themes: the enjoyment the pupils felt from being in nature and learning about it; the responsibility pupils felt for looking after nature; and thirdly, how these two aspects helped to contribute to a sense of better mental health and wellbeing. We conclude with a consideration of the potential for integrating what we have learnt within educational settings in the UK and more widely.

# **Keywords**

youth mental health, therapeutic landscapes, situated and embodied learning

# **Introduction**

The ‘Rooted in Nature’ (RiN) research project grew out of a concern about how the social and educational isolation imposed in the UK during the first year of the COVID-19 pandemic (March 2020-March 2021) would continue to pose an increased threat to the mental health of children and young people (hereafter referred to as "children"). During 2021, two of the authors (Catherine El Zerbi & Clare Woolhouse) joined an online research interest group concerned with youth mental health in the north of England and found they both had an interest in using arts-based research methods to improve how researchers communicate with children. On further discussion with other colleagues, the seeds for RiN were sown. These seeds sprouted when a serendipitous call was made by the UK Research and Innovation (UKRI) body to fund research on mobilising community assets to tackle health inequalities, particularly within communities that experience high levels of economic deprivation and marginalisation.

In this paper we start by outlining the background to the RiN study, the underpinning theoretical concept of ‘therapeutic landscapes’ and the co-created and arts-based data collection methods that included photo-elicitation led walking interviews, creative dilemma cafes, workshops and school based activities that included photo-elicitation interviews.

# **Background**

Recent research in England within the field of youth mental health has consistently raised concerns about how school closures during the COVID-19 pandemic amplified difficulties. The Mental Health and Young People Survey (NHS Digital, 2021), found that the 2017 pre-pandemic rate of mental health difficulties of one in nine children between the ages of 6 and 19, had increased to one in six by 2021 (see also Waite et al., 2021). Similarly, the UK charity MIND (2021) conducted a study of mental health in secondary schools and found that one and a half million children aged 11-18 were in urgent need of mental health services, and that over 95% of these children felt that their education had been affected by a lack of support for mental health (see also Liang et al.’s work, 2020).

The increasing challenges in youth mental health are set against a backdrop whereby the UK Government promised in 2017 that £300 million would be directed to improving mental health support within secondary schools (Department for Education (DfE), 2017). However, the implementation of this promise did not fully materialise and by 2021 less than £10 million had been spent (MIND, 2021). The concerns raised within the UK by MIND (2021), NHS Digital (2021) and Waite et al. (2021) were repeated globally; the mitigation efforts for addressing COVID-19, such as staying home, reducing social contact and school closures altered the lives of people all over the world (Kickbusch et al., 2020) and these in turn created mental health-related consequences in different national settings. Examples outside the UK context include Ciciurkaite et al.’s (2022) study of the US, Rajkumar’s (2020) review of research from China, Iran, Canada, Brazil, Singapore, India and Japan, Zhang et al.’s (2020) study in Zhongshan, in the Guangdong province of China, and Viner et al.’s (2021) review of the impact of school closures during COVID-19 in eleven countries, including China, Turkey, Brazil, Bangladesh and India, the US, Italy, Canada, Japan, and Spain.

While each national or regional setting may have had different dynamics or restrictions that interplayed with the complexities around whether it was the pandemic itself, or the sense of isolation created by restrictions, there are strong indications that during 2020-2021 pre-existing difficulties were amplified which produced greater challenges for young people’s mental health in the UK (see MIND, 2021; Newlove-Delgado et al., 2021; Samji et al., 2022). Furthermore, school closures meant that cross-professional relationships were disrupted, which according to Lee’s (2020) article about impact in Hong Kong and China, can exacerbate individuals’ mental health difficulties because engagement with school is viewed as an important coping mechanism for many children. Perhaps then, it is unsurprising that it is estimated that in the UK in 2022 two thirds of those with a mental health need were unable to access the support they needed (Young Minds, 2022).

In order to explore how schools and the mental health professionals that work with them can provide support that meets young people’s needs, in the remainder of this article we consider the potential therapeutic benefits of being outdoors in nature before discussing the case study example ‘Rooted in Nature’. While this study is based in a specific geographical location that is designated as an area of economic deprivation in North East England, we will also consider what the potential is for sharing the lessons learnt more widely.

# **Therapeutic Landscapes and the benefits of nature-based activities**

Over the past twenty years there has been an increasing interest in the use of outdoor spaces to promote mental health and wellbeing, alongside which a focus on related research has started to emerge. Bell et al. (2018, p.124) synthesises this work, undertaking an extensive review of 161 papers published between 2007-2016 to “explore the everyday experiences and practices that make such places health-enabling for individuals and communities”. In doing so Bell et al. (2018) define outside space as encompassing the potential to be a therapeutic landscape (TL), as a place that offers a sense of healing, whether that be in a built or natural environment. Bell et al. (2018, p.123) argue that practitioners and researchers developed the concept of TLs “to incorporate the health promoting (as well as healing) qualities of therapeutic spaces and the therapeutic value of everyday spaces. These included both aesthetic qualities and more imperceptible social networks offering a sense of security and inclusion”. Bell et al. (2018) continue, indicating that the conceptualisation and use of TLs can be understood as playing a valuable role in sensitising and encouraging researchers to critically engage with the complexities of how these spaces are experienced. However, it is argued that the influence of such work has failed to gain prominence within policy circles in the UK, possibly due to a lack of high-quality research evidence (Bell et al., 2018, p.128). This lack of engagement at a policy level has shifted more recently with the UK Government and health professionals recognising the importance of outside spaces for promoting mental health. In the guidance document: *Social prescribing: applying all our health* (UK Government, 2019) it is noted that the use of non-clinical settings for promoting health and wellbeing, tackling isolation and loneliness, can be accessed via health institutions through social prescribing which can build social connections through community-centred approaches to arts activities, group learning, walking, singing or gardening.

The potential benefits for mental health of utilising TLs has been reiterated by Birch et al. (2020) who used interviews and creative arts workshops to explore the value of urban nature for the mental health and wellbeing in a northern city of the UK. Birch et al. (2020), worked with 24 young people aged 17–27 years, nine of whom had lived experience of mental health difficulties. The young people involved expressed how their urban nature encounters were experienced as accepting and relational, offering a stronger sense of self; feelings of escape; connection and care with the human and non-human world (Birch et al., 2020, p.6-9). Doughty et al. (2022) conducted similar research in the Netherlands, also utilising creative methods to explore the use of local built environments. Doughty et al. (2022, p.662) indicated that “the pandemic has created new (micro-)geographies of healing and respite” arguing that outside space had become an important enabler of everyday wellbeing. A view supported in a study of over 6,000 children in Iceland, published by Thorstein et al. (2023), which noted that the benefits for children of ‘outdoors time’ included improved general health, but also positive developments for children’s peer relationships as well as their relationships to nature.

The work cited so far has identified the potential benefits of activities utilising outside spaces but has also called for greater engagement via research with a wider range of personalised complex experiences to better understand the health benefits of nature-based programmes. In some part our project responds to this call by adopting a practice-focused stance situated within education and community contexts.

# **The Rooted in Nature Project**

The ‘Rooted in Nature’ (RiN) project is a co-produced interdisciplinary study drawing together academics working in the fields of Health and Education to research with community partners based in Middlesbrough, North East England. In this project a diverse group of local young people used creative methods to explore what they most value about participating in local nature-based activities and how such activities may improve health and wellbeing. The data set was gathered via photo-elicitation with children and young people and the adults that work with them. This included walking interviews with nature-based practitioners working for a social prescribing service and with the service users. In both cases the interviewees took photographs as they walked, and discussing them with an embedded researcher along the route and in a follow up interview. The team also organised creative dilemma cafes within which key stakeholders, such as service users, practitioners, academics and local policy makers, discussed the collated photographs to explore experiences of TLs and views about current services. The embedded researcher also conducted a number of facilitated school workshops (discussed in more detail below). A scoping review was also conducted around the activities of organisations, community groups and practitioners across North East England who were working with children to support mental health. These various methods of data collection were designed to help us understand the value of nature-based programmes for mental health and identify the requirements for effective infrastructure management and contextual adaptation for scaling-up across the North East and North Cumbria (NENC) region. The researchers also wanted to engage stakeholders to establish the conditions for sustainability, specifically to identify gaps, constraints and opportunities for integrating nature-based programmes into local health and education systems in the future.

## **Respecting pupil’s voices utilising a creative approach**

In this article we focus upon the school-based workshops that were one part of the project.

A multisensory approach (Pink, 2011) was adopted to developing school eco gardens through researcher facilitated workshops that utilised creative visual materials, spatiality and tactile experience to better access the agency of participants. These sessions were designed and delivered by a community partner and conducted in a school with a class of secondary aged children and the Deputy Principal. During the research the data collection involved taking photographs of various nature-based activities, which included planting and tending the garden areas, building bird feeders and bug hotels, identifying unknown flora and fauna and documenting this work in an individual outdoor learning journal.

Such a multisensory methodology can be framed as belonging within an interpretivist paradigm (Cohen et al., 2018) since visual and tactile materials were utilised to enable participants to share what they thought or felt about their engagement with the activities. Photo-elicitation (Harper, 2002; Woolhouse, 2019) was a key creative method, it can be utilised as an appropriate, familiar and accessible way for people to express themselves and can be a route for accessing opinions in a different form. As Burns et al. (2023, p.26) point out following research in Iranian schools; “inclusion of photographs, and the detailed reflection upon these, adds up to a much more nuanced and comprehensive account.”

Using photographs in RiN was particularly beneficial as some of the children involved had English as an additional language and found that using photographs assisted them in sharing their points of view. Within the school workshops this sharing of opinions encompassed inviting the children to produce photographs of RiN activities and these, alongside the learning journals, were utilised as prompts during discussions that followed. It was felt that this approach offered a suitably ethical way to access life experiences, enlighten us as researchers and foreground participants’ wisdom. In particular we wanted to access the views of young people which have been largely absent from existing literature on therapeutic landscapes, social prescribing and mental health, since previous research has tended to focus either on younger children or older people (Bell et al., 2018; Doughty, 2018; Genter et al., 2015; Howarth et al., 2021; Zeivots, 2019) rather than teenagers.

For the RiN research it was felt it was essential to refocus upon youth mental health in the hope this could offer routes for addressing concerns in a scalable way within schools and other educational settings as well as within community services. To explore this potential, in the following section we analyse the data collected from the detailed photo-elicitation interviews with six of the children who were involved in school-based nature activities. Three interviews were conducted by the second and third authors of this paper (CEZ & CMT) with six pupils in friendship pairs - Sam and JJ, Taylor and Alex, Leli and Tre (Pseudonyms have been used to anonymise the data). The researchers followed the pre-approved ethical protocols, asking questions from the topic guide, as required. During the interviews the children were asked to consider what they felt were the benefits of outdoor learning.

# **Understanding children’s views of therapeutic nature-based activities**

Three key themes to emerge from the school-based workshops are discussed in this section: firstly the enjoyment the pupils felt from being in nature and learning about it; secondly the responsibility they felt for looking after nature; and thirdly, how these two aspects helped to contribute to a sense of better mental health and wellbeing.

## ***The importance of having fun***

In the RiN project, the young people themselves stressed the importance of becoming involved in nature-based activities at school, noting the value in collecting and observing nature, learning about it and having fun at the same time. Comments shared during discussions included:

It’s a fun club to go to. Like Sam said, you can do it any time of the year. When the seasons change, you might pick up some stuff from winter, perhaps spring. Like you could put different categories in your book, like autumn, summer, spring. … It could help people get into wildlife more and enjoy it a bit more, than just sitting in your house, playing games. So yes, it’s something fun to do when you're out and about. JJ

This one, as well [points at photograph of pupils in the school eco garden], I think it’s just nice because I took loads of samples. And it’s just nice because it reminds me how much fun we had when we were taking pictures and talking and learning about them. Leli

Tre picked up on the theme of enjoying being in and learning about nature, and noted the importance of engaging with the world around them rather than being focused on a mobile phone when the researcher asked how being involved in the eco garden activity made them feel:

It was pretty fun, getting to experience nature without being on your phones all the time. And it definitely gave you a new perspective on how the world works. Because normally you don’t pay attention to that one flower that looks really nice, you don’t really care. But when you're not on your phone and you're just looking at it a different way, it gives you a new perspective on things. … So in my opinion, nature is more about the actual plants. Not just plants, but the outside, it really depends where you're doing it and how you're doing it. Because [with] sports, you're not immersed in nature, you're immersed in the sport. Which isn't bad, but it’s different.	Whereas this [points to photos of eco garden – See Figure 1] it’s having fun.

Insert Figure 1 – Developing the eco garden.

The importance of ‘joy’ and ‘play’ in learning settings cannot be understated and yet can sometimes be left behind when children leave primary school. Secondary school students usually experience less opportunity for play and joyful learning; for example, preparing exam skills takes over from the joy of solving educational puzzles. Yet, play induces positive chemical reactions in our bodies, releasing dopamine, oxytocin and serotonin which leads to ‘habit formation and reward-seeking behaviours’ (Koeners & Francis, 2020, p.145) therefore increasing students’ desires or willingness to engage with learning. Indeed, Li et al. (2020) note that by weaving play and joy into the learning experience, students experience increased motivation and desire to interact socially, which can increase the students’ ability to memorise information and maximise their attention span as well as enhance peer friendships (see also Thorstein et al., 2023). Of course, ensuring learning is fun will also create a positive atmosphere which reduces the stress often found in exam classes in different national contexts.

## ***Looking after nature***

The children highlighted how the experiences of building the eco gardens demonstrated to them that they were grounded in, and responsible for, the natural environments around them. It was felt that they were “part of something larger” (Howarth et al., 2021, abstract) and could contribute to their communities by enhancing their local environment. For example, Sam mentioned;

You can watch the plants, fruit or vegetables grow, as it goes through time. Then you can watch it go from small to really big. And I think it’s just a really nice process to watch. … It could be maybe a good stress relief for people, if they just wanted to be outside. Instead of just sitting outside, you could actually be doing something, that’s really helpful for the environment too.

Building on this idea further, Sam emphasised the importance of individual responsibility for the natural environment as they discussed photographs of the bird feeders they had made (see Figures 2 & 3):

I’ve done something that’s actually useful. Because sometimes, when you make stuff, it might not be used, mightn’t it? But it makes you feel proud of what you’ve done, that you’ve made something that can help the animals. And it just looks pretty in the [school] garden too. … So this is my journal. And on the front, it says you need to keep the rare things safe. Because you know how there are some animals that are out there that are very rare, there are not many of them left, they're endangered? You need to keep them safe, keep them in a good habitat.

Insert Figures 2 & 3: Building bird feeders and bug houses

This participant repeated the notion of being 'useful' in the small ways in which they could help nature, and the desire to do so, was clear. Their comments referred to a sense of pride at helping animals and conservationism was identified as important to the participants. The process of learning about nature by being in it and engaging in creative activities (see Figures 4 & 5 for further examples) was explicitly identified as enjoyable. Participants also noted how such activities could inform their understandings around future responsibilities for conserving nature.

Insert Figures 4 & 5: Creating a Journal

This view echoes the point made by Restall and Conrad (2015) and Zylstra et al. (2014), that there are resonances between human welfare and the welfare of the natural world. For example, the children stated;

This is in my journal. … I think it’s a good way to get into nature… Get your findings and put them in a book. And you can just use the nature, and it’s just a good way to enjoy [it] … I feel like it’s a good thing to do, because you could take it back and look at what we’ve done, to make improvements to wildlife and nature. If there’s anything that you see that you think is really good, then you can take that, look back at it and see what we’ve done and what you can build on. (JJ)

[Other young people would enjoy engaging in the activities because] then they will be more motivated in nature. So it’s good for their health and nature’s health. Because then, in the future, they can help nature, clean the entire earth. (Taylor)

I took six photos. For this photo, it’s one lone tree in a barren wasteland of zero trees. And it’s kind of showing how it’s like a survivor. It just interested me how this tree has survived and all the other trees died. It’s kind of symbolic of people really, how we have to be resilient and not give up, even if everyone else does. It just looks pretty cool in this barren wasteland, which is kind of depressing. A symbol of hope. (Tre)

The suggestion that children are aware of their connection to the wider world and recognise existing threats to their local environments and more broadly to the global climate has been recognised (Charles et al., 2018; Howarth et al., 2021; Ives et al., 2017). Therefore, engaging children in looking after nature on a small scale, for example in a school eco garden, offers some hope that they will develop a sense of pride and ownership that can be taken outside the school gates. As Ives et al. (2017) argue, childhood is a perfect time for individuals to develop a sense of emotional, personal and respectful connection with nature which can lead to involvement in a more impactful social and environmental change. This is not just beneficial for the world at large, it would seem that the benefits of being involved with nature, being involved in the protection of nature personally, can be life enhancing as well as socially responsible (Larson et al., 2017).

## ***The therapeutic potential of using nature-based activities in schools***

The importance of having fun and being socially responsible were explicitly drawn together by the children in comments about the benefits for individual health and wellbeing. As already noted in response to Bell et al. (2018) and Howarth et al.‘s (2021) work, the therapeutic benefits of engaging in nature-based activities have been repeatedly stated. Such benefits came through in the comments offered by the children when they were interviewed. For example, Sam talked about a picture they had taken of a sycamore leaf:

If you watch them [the seeds] fall down, they’ll spin round and round. It’s just they're a nice thing to watch, and it will make you feel happy when you're watching it, knowing that you’ve done something that can actually make you feel happy.

When asked by the researchers if Tre went into nature with their family, they replied:

Sometimes I go by myself, sometimes I go … my family is pretty busy, so I do just go outside sometimes by myself. But sometimes I go with my friends, which is a bit better, because we get to chat.

Building on this idea, and reiterating the findings of Birch et al. (2020), the children were able to identify the bigger picture of how connectedness with nature can influence the physical and mental health of people:

[Being involved in the eco garden] will help my health because instead of really bad and horrible things, thinking about nice things, it will just help your mental health really. Because you’ll be more active. And even in lessons, you’d be listening more as well. … I read things about it online, and I didn’t really believe it. But after doing it myself a couple of times, it feels good to be like - not one with nature, but just observing the surroundings. And being fully immersed in where you are. Being mindful, I guess. (Leli)

It’s kind of like a detox, I guess, from being on your phones all the time. It’s just a bit like … It’s better than people would think. it makes you feel good, to see that [insects in the eco garden], I guess, and be immersed in it. It will make them feel good. Because if you're feeling down one day, you can go back and see these pictures of you having fun in nature and taking pictures of cool things, I guess. It will uplift your mood. … I would say, when you're on your phone 24 hours a day, you’ll have bad thoughts in your mind because of all the bad things that you might be watching. If you go outside, I don't know, even for five minutes or something, you’ll clear your mind, you’ll forget about everything you thought about. (Tre)

The comment above reiterates how immersion in nature can be an antidote to the almost constant presence of mobile phones. This demonstrates within the children’s personal testimonies how the act of being involved in a small scale eco-garden project can provide a space to reflect upon the experience and make links between engagement with nature and individual health. This view acknowledges the therapeutic benefits of being involved in outside spaces, supporting the argument made more than a decade apart by Milligan et al. (2004) and by Bell et al. (2018) that benefits for health can be experienced in different types of places, contexts and landscapes, including a space such as a local disused wasteland rather than the perhaps more expected green spaces. In this case, re-purposing unused land on school grounds had been a project of a particular teacher. As part of the RiN project one of the community partners who was the Teacher Project Lead for RiN in her school sought to breathe new life into the garden, which is now a valued school asset that was under-utilised. One of the take-away messages should be that local authorities could audit the extent to which schools and other community groups in the North East of England, and further afield, have under-utilised spaces such as this one. It is also worth considering what it would take to utilise them in the service of health and wellbeing interventions. This re-engagement with existing, unused areas of land is important because they are free and as Gesler (1992) points out, therapeutic landscapes can be any environment, natural or built so long as they have the potential to be experienced as healing. A view shared by Howarth et al. (2021, p.5) who highlight the potential of therapeutic landscapes “to provide a place of safety that can enable recovery and re-engagement”.

**Conclusion: How can the findings inform change in schools? What is needed next?**

The RiN research project sought to evaluate the features of nature-based activities in a school in North-East England by asking the children involved what they thought and felt about designing and maintaining a small scale eco garden within their school grounds. While we acknowledge the limitations of having a small and contained dataset, and sharing the thoughts of just six children, this does not negate the importance of their opinions and experiences. The intention of the authors is to continue by extending this work to engage a range of schools in order that the children’s views could be compared with others and shared more widely.

The identified therapeutic and other benefits of learning outside for young people indicate that alongside increased access to more formal opportunities for social prescribing, there should be opportunities within school for regular, interdisciplinary and cross-curricular nature-based learning outside the classroom. This view is particularly advocated by researchers working in Northern Europe: Lauterbach et al.’s (2024) work from Norway, Bølling et al, (2018) in Denmark, Harris (2021) in the UK and van Dijk-Wesselius et al. (2020) in the Netherlands. Although, Waite’s (2020) comparison of school based outdoor learning from nineteen countries and areas also highlights the need for nuanced educational, cultural and material contextual sensitivity when embedding outdoor learning within school curricula. As has been pointed out by the various studies cited, the capacity for schools and health providers from a range of national contexts to enable this is restricted by a lack of long-term, strategic and sustainable funding that invests in local knowledge and produces legacies for nature-based programmes.

There is a concern that while many young people do not receive support from school for their mental health, many do not want it. As MIND (2021, p.47) point out: “Less than four in ten (38%) young people surveyed said they had received support from school for their mental health and over a third (36%) of young people said that they did not want mental health support from their school”. This might be related to a reluctance from students to share details with a teacher rather than not wanting support with mental health, and Hertz et al. (2022, p.61) suggest, what is needed is “a comprehensive, coordinated, multidisciplinary approach to promoting student mental health and well-being" to include “diverse partners across education, health, and mental health to plan for and meet the needs of students and their families”.

This being the case, maybe it would be beneficial for schools working with other community groups to offer a more neutral space for learning where the explicit focus is around learning in and about nature, sustainability or the environment. This stance aligns with a number of the UN sustainability goals, particularly Goal 13: Climate Action, Goal 3: Good Health and Wellbeing and Goal 11: Sustainable Cities and Communities (UN, 2015). As this paper outlines, having the opportunity to be outside learning with peers and others brings global issues to a personal level, whereby discussion around experiences of nature and wellbeing are encouraged, because “the notion of therapeutic landscapes can support social interaction and ultimately create therapeutic spaces” (Howarth et al., 2021, p.6). One potential route for enabling this might be a more integrated and transformative approach whereby young people themselves co-design and co-deliver nature-based work, guiding learning opportunities and peer-lead activities. As Howarth et al. (2021, p.7) notes, “the success and sustainability of future nature-based projects means adopting a transformative approach that tackles a system predicated on a pathogenic rather than wellness model”. In turn this could lead to a more varied and permanent range of opportunities to be offered to young people within and outside schools, with a focus on forms of learning about nature that include exploration, adventure, discovery and creativity.

## **References**

Bell, S. L., Foley, R., Houghton, F., Maddrell, A. & Williams, A. M. (2018). From therapeutic landscapes to healthy spaces, places and practices: A scoping review. *Social science & medicine*, *196*, 123-130. <https://www.sciencedirect.com/science/article/pii/S2666558122000549>

Birch, J., Rishbeth, C. & Payne, S. R., (2020). Nature doesn't judge you–how urban nature supports young people's mental health and wellbeing in a diverse UK city. *Health & Place*, *62*, 102296. <https://www.sciencedirect.com/science/article/pii/S135382921931158X>

Bølling, M., Otte, C. R., Elsborg, P., Nielsen, G., & Bentsen, P. (2018). The association between education outside the classroom and students’ school motivation: Results from a one-school-year quasi-experiment. *International Journal of Educational Research*. 89, 22–35. <https://www.sciencedirect.com/science/article/pii/S0883035518300120>

Burns, E. A., Manouchehri, B. & Davoudi, S. (2023). A narrative photographic approach: opening day ceremonies for Iran’s Nature Schools. *Journal of Outdoor and Environmental Education* 26, 23–47. <https://link.springer.com/article/10.1007/s42322-022-00111-3>

Charles, C., Keenleyside, K., Chapple, R., Kilburn, B., van der Leest, P. S., Allen, D., & Camargo, L. (2018). *Home to us all: How connecting with nature helps us care for ourselves and the earth*. Minneapolis, MN: Children and Nature Network. <https://repository.derby.ac.uk/item/95424/home-to-us-all-how-connecting-with-nature-helps-us-care-for-ourselves-and-the-earth>

Ciciurkaite, G., Marquez‐Velarde, G., & Brown, R. L. (2022). Stressors associated with the COVID‐19 pandemic, disability, 318 and mental health: Considerations from the Inter mountain West. *Stress and Health*, 38(2), 304–317. <https://onlinelibrary.wiley.com/doi/full/10.1002/smi.3091>

Cohen, L., Manion, L. & Morrison, K. (2018). *Research methods in education* (8^th^ edition). Routledge. <https://doi.org/10.4324/9781315456539>

Department for Education (DfE). (2017). *Transforming children and young people’s mental health provision: a green paper*. <https://assets.publishing.service.gov.uk/government/uploads/system/uploads/attachment_data/file/664855/Transforming_children_and_young_people_s_mental_health_provision.pdf>

Doughty, K. (2018). Therapeutic landscapes. In Howard, P., Thompson, I., Waterton, E. & Atha, A. (Eds.) *The Routledge companion to landscape studies* (pp. 341-353). Routledge.

Doughty, K., Hu, H. & Smit, J. (2022). Therapeutic landscapes during the COVID-19 pandemic: increased and intensified interactions with nature, *Social & Cultural Geography*, 24(3-4), 661-679. <https://www.tandfonline.com/doi/full/10.1080/14649365.2022.2052168>

Genter, C., Roberts, A., Richardson, J. & Sheaff, M. (2015). The contribution of allotment gardening to health and wellbeing: A systematic review of the literature. *British Journal of Occupational Therapy*, *78*(10), 593-605. <https://doi.org/10.1177/0308022615599408>

Gesler, W. (1992). Therapeutic landscapes: Medical issues in light of the new cultural geography, *Social Science & Medicine*, 234(7) pp.735-746. <https://www.sciencedirect.com/science/article/abs/pii/0277953692903603>

Harper, D. (2002). “Talking about Pictures: a case for Photo-elicitation.” *Visual Studies*, 17(1), 13–26. <https://doi.org/10.1080/14725860220137345>

Harris, M. A. (2021). Growing among trees: A 12-month process evaluation of school based outdoor learning interventions. *Journal of Adventure Education and Outdoor Learning*. 23(3), 232-243. <https://doi.org/10.1080/14729679.2021.2001758>

Hertz, M. F., Kilmer, G., Verlenden, J., Liddon, N., Rasberry, C. N., Barrios, L. C. & Ethier, K. A. (2022). Adolescent mental health, connectedness, and mode of school instruction during COVID-19. *Journal of Adolescent Health*, *70*(1), 57-63. https://pubmed.ncbi.nlm.nih.gov/34930571/

Howarth, M., Lawler, C. & da Silva, A. (2021). Creating a transformative space for change: A qualitative evaluation of the RHS Wellbeing Programme for people with long term conditions. *Health & Place*, *71*, p.102654. <https://www.sciencedirect.com/science/article/pii/S1353829221001507>

Ives, C. D., Giusti, M., Fischer, J., Abson, D. J., Klaniecki, K., Dorninger, C. & von Wehrden, H. (2017). Human–nature connection: A multidisciplinary review. *Current Opinion in Environmental Sustainability*, 26–27, 106–113. <https://doi.org/10.1016/j.cosust.2017.05.005>

Kickbusch, I., Leung, G. M., Bhutta, Z. A., Matsoso, M. P., Ihekweazu, C. & Abbasi, K. (2020). COVID‐19: How a virus is turning the world upside down. *BMJ*, 369, Article m1336. [https://doi.org/10.1136/bmj. M1336 363](https://doi.org/10.1136/bmj.%20m1336%20363)

Koeners, M. P., & Francis, J. (2020). The physiology of play: Potential relevance for higher education. *International Journal of Play*, *9*(1), 143-159. <https://www.tandfonline.com/doi/full/10.1080/21594937.2020.1720128>

Larson, L., Bowers, E. & Stephens, L. (2017). Connection to nature and positive youth development. Estes Park, CO: Presented at Pathways 2017: Integrating Human Dimensions into Fisheries and Wildlife Management.

Lauterbach, G., Bølling, M. & Dettweiler, U. (2024). Education Outside the Classroom in Norway: The prevalence, provision, and nature of uteskole. *International Journal of Educational Research*, *125*, 102349. <https://www.sciencedirect.com/science/article/pii/S0883035524000363>

Lee, J. (2020). Mental health effects of school closures during COVID-19. *The Lancet Child & Adolescent Health*, *4*(6), 421. <https://www.thelancet.com/journals/lanchi/article/PIIS23524642(20)30109-7/fulltext>

Li, L., Gow, A. D. I. & Zhou, J. (2020). The role of positive emotions in education: A neuroscience perspective. *Mind, Brain, and Education*, *14*(3), 220-234. <https://psycnet.apa.org/doi/10.1111/mbe.12244>

Liang, L., Ren, H., Cao, R., Hu, Y., Qin, Z., Li, C. & Mei, S. (2020). The effect of COVID-19 on youth mental health. *Psychiatric quarterly*, *91*(3), pp.841-852. <https://pubmed.ncbi.nlm.nih.gov/32319041/>

Milligan, C., Gatrell, A. & Bingley, A. (2004). ‘Cultivating health’: therapeutic landscapes and older people in northern England, *Social Science & Medicine*, 58(9), pp.1781-1793. <https://pubmed.ncbi.nlm.nih.gov/14990378/>

MIND. (2021). *Not making the grade* *report: why our approach to mental health at secondary school is failing young people.* <https://www.mind.org.uk/media/8852/not-making-the-grade.pdf>

Newlove-Delgado, T., McManus, S., Sadler, K., Thandi, S., Vizard, T., Cartwright, C. & Ford, T. (2021). Child mental health in England before and during the COVID-19 lockdown. *The Lancet Psychiatry*, *8*(5), pp.353-354. <https://pubmed.ncbi.nlm.nih.gov/33444548/>

NHS Digital. (2021). *Mental Health of Children and Young People in England 2021 – wave 2 follow up to the 2017 survey.* <https://digital.nhs.uk/data-and-information/publications/statistical/mental-health-of-children-and-young-people-in-england/2021-follow-up-to-the-2017-survey>

Pink, S. (2011). Multimodal, multisensory and ethnographic knowing: social semiotics and the phenomenology of perception. *Qualitative Research*, 11 (3) pp.261-276. <https://doi.org/10.1177/1468794111399835>

Rajkumar, R. P. (2020). COVID‐19 and mental health: A review of the existing literature. *Asian Journal of Psychiatry*, 52, 395 Article 102066. <https://doi.org/10.1016/j.ajp.2020.102066>

Restall, B. & Conrad, E. (2015). A literature review of connectedness to nature and its potential for environmental management. *Journal of Environmental Management*, 159, 264–278. <https://doi.org/10.1016/j.jenvman.2015.05.022>

Samji, H., Wu, J., Ladak, A., Vossen, C., Stewart, E., Dove, N. & Snell, G. (2022). Mental health impacts of the COVID‐19 pandemic on children and youth–a systematic review. *Child and adolescent mental health*, *27*(2), 173-189. <https://acamh.onlinelibrary.wiley.com/doi/full/10.1111/camh.12501>

Thorsteinsson, J. F., Arnarsson, Á. & Jónasson, J.T. (2023). How ‘outdoors time’ transforms the social relationships of children in Iceland. *Journal of Outdoor and Environmental Education*, 26, 227-246. <https://link.springer.com/article/10.1007/s42322-023-00123-7>

UK Government announcement. (January 2019). https://www.gov.uk/government/publications/social-prescribing-applying-all-our-health/social-prescribing-applying-all-our-health#social-prescribing

United Nations (2015) The 2030 Agenda for Sustainable Development. Retrieved December 19, 2024, from <https://sdgs.un.org/2030agenda>

van Dijk-Wesselius, J. E., Van den Berg, A. E., Maas, J. & Hovinga, D. (2020). Green schoolyards as outdoor learning environments: Barriers and solutions as experienced by primary school teachers. *Frontiers in Psychology*, *10*, p.2919. <https://www.frontiersin.org/journals/psychology/articles/10.3389/fpsyg.2019.02919/full>

Viner, R., Russell, S., Saulle, R., Croker, H., Stansfeld, C., Packer, J., Nicholls, D., Goddings, A. L., Bonell, C., Hudson, L. & Hope, S. (2021). Impacts of school closures on physical and mental health of children and young people: a systematic review. *MedRxiv.* <https://www.medrxiv.org/content/10.1101/2021.02.10.21251526v1>

Waite, S. (2020). Where are we going? International views on purposes, practices and barriers in school-based outdoor learning. *Education Sciences*, *10*(11), 311. <https://www.mdpi.com/2227-7102/10/11/311>

Waite, P., Pearcey, S., Shum, A., Raw, J. A., Patalay, P. & Creswell, C. (2021). How did the mental health symptoms of children and adolescents change over early lockdown during the COVID‐19 pandemic in the UK?. *JCPP advances*, *1*(1), p.e12009. <https://pubmed.ncbi.nlm.nih.gov/34485988/>

Woolhouse, C. (2019). Conducting photo methodologies with children: framing ethical concerns relating to representation, voice and data analysis when exploring educational inclusion with children. *International Journal of Research and Method in Education. 42*(1) P.3-18. <http://dx.doi.org/10.1080/1743727X.2017.1369511>

Young Minds (2022). *Coronavirus: Impact on young people with mental health needs.* Retrieved December 19, 2024, from <https://www.youngminds.org.uk/about-us/reports-and-impact/>

Zeivots, S. (2019). Escaping to nature to learn: emotional highs of adult learners. *Journal of Outdoor and Environmental Education* 22, 199–216. <https://www.researchgate.net/publication/336348432_Escaping_to_nature_to_learn_emotional_highs_of_adult_learners>

Zhang, J., Lu, H., Zeng, H., Zhang, S., Du, Q., Jiang, T. & Du, B. (2020). The differential psychological distress of populations affected by the COVID‐19 pandemic. *Brain, Behavior, and Immunity*, 87, 49–50. <https://pmc.ncbi.nlm.nih.gov/articles/PMC7156946/>

Zylstra, M. J., Esler, K. J., Knight, A. T. & Le Grange, L. L. (2014). Connectedness as a core conservation concern: An interdisciplinary review of theory and a call for practice. *Springer Science Reviews*, 2, 119–143. <https://link.springer.com/article/10.1007/s40362-014-0021-3>
